# Normal Healthy Aging Does Not Alter Large‐Scale Brain Network Stability

**DOI:** 10.1002/alz70856_101148

**Published:** 2025-12-25

**Authors:** Bardiya Ghaderi Yazdi, Peter Chernek, Seyed Hani Hojjati, Sindy Ozoria, Jenseric Calimag, Qolamreza R Razlighi

**Affiliations:** ^1^ Weill Cornell Medicine, New York City, NY, USA

## Abstract

**Background:**

Within‐network functional connectivity (FC) is commonly used to evaluate neurologic and psychiatric diseases. Understanding FC patterns in young and old healthy subjects is critical to distinguishing healthy conditions from pathological. Here, we investigate age‐related alterations within the brain's large‐scale network connectivity using resting functional magnetic resonance imaging (rfMRI), and FC analysis of cognitively unimpaired (CU) individuals compared to young healthy controls (HC) controlling motion and atrophy.

**Method:**

We analyzed rfMRI scans from 60 (50% females) HC (27.95±5.3) and 191 (50% females) CU (68.6±5.7) from the Quantitative Neuroimaging Laboratory Repository (QNLR) using our fMRI pipeline (i.e. geometric distortion correction, slice timing correction, realignment, AROMA motion correction, low‐pass filtering, scrubbing, residualizing with white‐matter/ventricles or motion parameters, and spatial normalization using ANTS.) Using the 200*200 Schaefer atlas and a normalized subject‐specific binary mask of gray matter, we obtained the average regional time series of 200 nodes to compute the inter‐regional connectivity using Pearson's Correlations. The mean correlations within each network gave the connectivity level for each participant. These networks include the Visual Network, Sensorimotor Network, Dorsal Attention Network, Ventral Attention Network, Limbic Network (LIM), Frontoparietal Network, and Default Mode Network. QNLR also holds data for amyloid accumulation in the brain, using an 18F‐Florbetaben positron emission tomography scan. Based on visual reading and the presence of abnormal amyloid deposition we categorized subjects to amyloid positive and negative.

**Result:**

There was no significant differences between HC and CU in the connectivity of the brain's large‐scale networks. However, HC had higher FC in LIM (*p*‐value=0.02) when controlling for motion. Including only amyloid‐negative CU (139 participants) did not change the results; and LIM's FC (*p*‐value=0.02) remained significantly higher in HC. Excluding participants with any motion (20 HC and 41 CU) did not change the results, except there was a partially significantly higher LIM's FC in CU. (*p*‐value=0.056) None of these significant findings in LIM survived Bonferroni correction.

**Conclusion:**

This study suggests that normal healthy aging does not alter large‐scale brain within network stability. While some studies have reported a decline in rs‐FC across healthy aging, this might be partly due to differential motion and/or brain atrophy.

